# Risk of adenoma recurrence after polypectomy in patients younger than 50 years vs. 50 years old and over with diminutive or small adenomas

**DOI:** 10.3389/fonc.2022.823263

**Published:** 2022-10-25

**Authors:** Sicheng Cai, Huiying Shi, Mengke Fan, Qin Zhang, Rong Lin

**Affiliations:** ^1^ Department of Gastroenterology, Union Hospital, Tongji Medical College, Huazhong University of Science and Technology, Wuhan, China; ^2^ Department of Pathology, Union Hospital, Tongji Medical College, Huazhong University of Science and Technology, Wuhan, China

**Keywords:** adenoma, colorectal cancer, polyp, age, risk factor, surveillance

## Abstract

**Background and aims:**

Current studies have shown that polyp recurrence occurs after colonic adenomas polypectomy (AP), but the difference in recurrence risk between patients in patients older than 50 years and younger than 50 years has not been clearly studied.

**Methods:**

490 patients after AP were enrolled in the study. The patients were classified according to age (<50 years old or ≥50 years old), and then further categorized according to the baseline adenoma characteristics: Group 1: 1–2 non-advanced adenomas (NAAs) 1–5 mm in size; Group 2: ≥3 NAAs, 1–5 mm; Group 3: 1–2 NAAs, 6–9 mm; Group 4: ≥3 NAAs, 6–9 mm; and Group 5: advanced adenomas.

**Results:**

During a mean follow-up interval of 2.52 years (2.51 years for ≥50 years old and 2.55 years for patients <50 years old), NAA recurrence was detected in 147 patients (30.0%). Overall, the hazard ratio (HR) for NAA recurrence after AP was higher in patients ≥50 years old than that in patients <50 years old (HR, 1.774, *P* = 0.003). For patients <50 years old, HRs (Group 2-5 vs. G1, respectively) for NAA recurrence were 0.744 (*P* = 0.773), 3.885 (*P* = 0.007), 5.337 (*P* = 0.003), and 3.334 (*P* = 0.015). For patients ≥50 years old, HRs (Group 2-5 vs. G1, respectively) for NAA recurrence were 1.033 (*P* = 0.965), 1.250 (*P* = 0.405), 2.252 (*P* = 0.015), and 1.887 (*P* = 0.009). For G1, the risk of NAA recurrence was significantly higher in patients ≥50 years old (HR, 2.932, *P* = 0.011) than that in patients <50 years old; for G2–G5, the risk was similar in the two age groups (*P* > 0.05).

**Conclusions:**

For patients <50 years old with less than 3 NAAs that are 1–5 mm in size, the recurrence rate of NAA is less than that of patients ≥50 years old with the same index colonoscopy findings. When the adenomas are ≥5 mm, or their number exceeds 3, they have similar recurrence risk as that for patients ≥50 years old.

## 1 Introduction

Colorectal cancer (CRC) was estimated to be the fifth most commonly diagnosed cancer and a leading cause of death related to cancer worldwide in 2020 (1). Colonoscopy and polypectomy are routine methods for CRC and precancerous lesion screening to prevent CRC. As evidenced by clinical practice, removal of adenomatous polyps reduces the incidence and mortality rate of CRC (2, 3). Adenoma is one important type of precancerous lesions, and it is thought that almost 90% of CRC develops from adenoma (4). Therefore, it is recommended that adenomatous polyp be removed immediately after identification during colonoscopy (5). Nevertheless, the recurrence rate of adenoma is very high, reaching nearly 50% during follow-up (6). This suggests that patients with adenomas should be followed up in accordance with the risk of adenoma recurrence and metachronous CRC development.

According to the current guideline (7), patients ≥50 years old should be stratified based on the polyp baseline number, size, and histology during post-polypectomy surveillance. Specifically, tubular adenomas smaller than 10 mm and without high-grade dysplasia are classed as non-advanced adenomas (NAAs), and one to two NAAs that are smaller than 10 mm are classed as low-risk adenomas (7). Timely intervention and follow-up are essential to improve the prognosis of patients with NAAs. Several studies have demonstrated a major protective effect of polypectomy in patients with NAAs (8, 9). According to these studies, after polypectomy, the risk of CRC in patients with NAAs is lower than that in the general population. But the difference in recurrence risk between patients older than 50 and those younger than 50 has not been clearly elucidated (10, 11). This makes choosing an appropriate surveillance interval for these patients very difficult. Further, analysis of subcategories of NAAs of different sizes (1–5 mm vs. 6–9 mm) in individuals >50 years old revealed different risks of developing metachronous advanced neoplasia associated with NAAs of different sizes (12–18). It is not known whether the same is true for individuals younger than 50 years.

Accordingly, in the current study, we compared the risk of adenoma recurrence after NAA polypectomy in patients <50 years old and those ≥50 years old, to determine whether the currently recommended surveillance intervals are also suitable for patients <50 years old.

## 2 Methods

### 2.1 Study population

Patients undergoing colonoscopy from January 2012 to January 2020 at the Endoscopic Center of Wuhan Union Hospital (Wuhan, China) were considered for the study. Only patients who had undergone polypectomy of at least one polyp and were followed up for more than 1 year were included. And all the patients involved in this study were with Boston bowel preparation score greater than 6.

The exclusion criteria were: all resected polyps pathologically confirmed to be non-adenomatous; a history of CRC, inflammation bowel disease, schistosomiasis, and previous resection of any part of the colon; diagnosed with CRC, irritable bowel disease, and schistosomiasis at an index colonoscopy; poor bowel preparation; and lack of clinical information or histologic information on the polyps.

The endoscopic findings and histologic results were based on well-established electronic medical records. Data, including an identifier, sex, age, and polyp number, size, and histology were collected. All endoscopic reports and pathologic reports were manually reviewed by experienced endoscopists and pathologists.

### 2.2 Colonoscopy and histologic examination

All colonoscopies were performed using Olympus (Tokyo, Japan) CF-Q260 or CF-Q290 by experienced endoscopists. Polyp number and size were determined during the colonoscopy (11, 19, 20). Polyp size was determined after resection or using standard clinical practices, such as open biopsy forceps method. Polypectomy was carried on through argon plasma coagulation (APC), cold or hot snare polypectomy, endoscopic mucosal resection (EMR) or endoscopic submucosal dissection (ESD), where the polypectomy regimen was determined by endoscopist according to the actual condition of the patient. All collected specimens were carefully histologically investigated by pathologists.

### 2.3 Measurement and definition

Adenomas were stratified as follows: diminutive adenoma, 1–5 mm in diameter; small adenoma, 6–9 mm in diameter; advanced adenoma (AA), ≥10 mm in diameter, with tubulovillous or villous histology, or with high-grade dysplasia (7). Advanced neoplasia (AN) referred to the occurrence of either AAs or CRC. Serrated adenomas were excluded from consideration.

Patients were classified into two groups according to age, i.e., <50 years old (n = 163) and ≥50 years old (n = 327), and further sub-divided into five groups according to the number, size, and histology of polyps: Group 1 (G1), 1–2 diminutive NAAs; Group 2 (G2), 3 or more diminutive NAAs; Group 3 (G3), 1–2 small NAAs; Group 4 (G4), 3 or more small NAAs; and Group 5 (G5), AAs. The size of the adenoma was determined for the largest of the several present.

### 2.4 Statistical analysis

Baseline characteristics were compared using Chi-squared test, for categorical variables, and ANOVA, for continuous variables. Hazard ratios (HRs) for metachronous AN and adenoma recurrence were calculated using Cox proportional hazards regression model with 95% confidence interval (CI). Disease-free survival probabilities were determined using Kaplan–Meier method and survival curves were compared by log-rank test. All reported *P*-values are two-tailed, and *P* < 0.05 was considered to be statistically significant. All statistical analyses were performed using R software version 4.0.3, packages “survival” and “survminer” (21, 22).

## 3 Results

### 3.1 Patient characteristics

**Figure 1:**
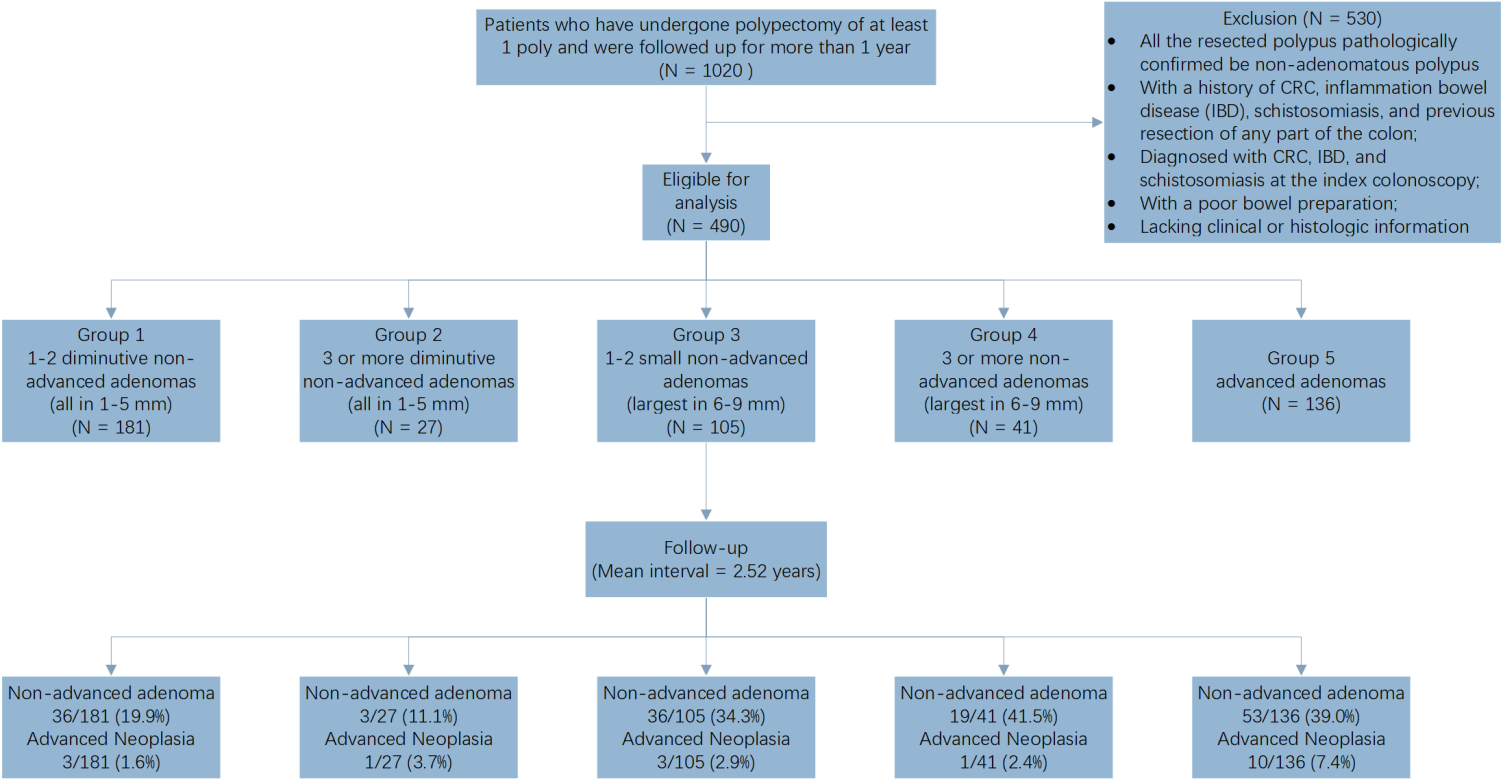
Study workflow.

The study workflow is shown in **Figure 1**. After initial patient screening using the inclusion and exclusion criteria, 490 patients were eligible for inclusion in the study. The patients were stratified per age, and then into predefined risk groups, according to the size, number, and histology of polyps. The demographic characteristics of patients included in the study at an index colonoscopy are shown in **Table 1**. The mean age was 54.1 ± 10.9 years; 29.8% (146/490) of the patients were female. The sex distribution did not differ significantly between all groups. The mean interval between the colonoscopy and surveillance was 2.52 ± 1.25 years (median: 2.18 years; range: 1.52–3.32 years), and the mean interval was 2.51 years for patients ≥50 years old and 2.55 years for patients <50 years old. The clinical findings of the surveillance are summarized in **Table 2**. During the follow-up, advanced neoplasia was rare, and found in only 18 patients (3.7%); NAA recurrence was more frequent, and detected in 147 patients (30.0%).

**Table 1:** Demographic characteristics of patients.

		1-2 diminutive NAAs	1-2 small NAAs	≥3 diminutive NAAs	≥3 small NAAs	AAs	*P*
**n**	Overall	181	27	105	41	136	
	<50yr	67	16	26	14	40	
	≥50yr	114	11	79	27	96	
**Age** **[mean (SD)]**	Overall	53.77 (10.15)	47.26 (11.17)	64.67 (11.25)	54.10 (11.35)	55.46 (11.12)	0.010
	<50yr	43.33 (5.38)	40.44 (8.42)	39.23 (6.56)	41.93 (7.58)	42.40 (5.89)	0.063
	≥50yr	59.91 (6.64)	57.18 (5.95)	59.75 (7.04)	60.41 (6.95)	60.90 (7.73)	0.493
**Gender** **[Female (%)]**	Overall	61 (33.5)	10 (37.0)	25 (23.8)	10 (24.4)	40 (29.4)	0.357
	<50yr	23 (34.3)	5 (31.2)	6 (23.1)	2 (14.2)	11 (27.5)	0.570
	≥50yr	38 (33.3)	5 (45.5)	19 (24.1)	8 (29.6)	29 (30.2)	0.531
**Surveillance interval** **[mean (SD)]**	Overall	2.52 (1.25)	1.95 (0.84)	2.55 (1.27)	2.50 (1.19)	2.61 (1.31)	0.170

NAA, Non-advanced adenoma; AA, Advanced adenomas; SD, Standard deviation.

**Table 2:** Pathologic findings during the surveillance.

		N (%)
**Advanced** **neoplasia**		18 (3.7)
	Cancer	9 (1.8)
	Adenoma ≥10 mm in size	0 (0)
	Adenoma with tubulovillous histology	8 (1.6)
	Adenoma with villous histology	1 (0.2)
**Non-advanced** **adenoma**		147 (30.0)

### 3.2 Risk of NAA recurrence after resection of diminutive vs. small adenomas in different age groups

The cumulative incidence of NAA was compared across different age groups and the polyp size–number groups (**Table 3**). The risk of NAA recurrence was significantly higher in patients aged ≥50 years than that in patients aged <50 years [HR, 1.77 (95% CI, 1.21–2.60), *P* = 0.003]. Further, the risk in the G3, G4, and G5 group was significantly lower than the individual risk in the G1 group [HR, 1.75 (95% CI, 1.11–2.78), *P* = 0.017, G3 vs. G1; 2.90 (95% CI, 1.66–5.06), *P* < 0.001, G4 vs. G1; and 2.32 (95% CI 1.52–3.54), *P* < 0.001, G5 vs. G1), but the risk in G2 group was similar to that in the G1 group [HR, 0.71 (95% CI, 0.22–2.31), *P* = 0.570]. This suggests that older age, large polyp size, and the presence of numerous polyps are potential risk factors for the recurrence of NAA after polypectomy.

**Table 3:** Risk of non-advanced adenoma recurrence according to the colonoscopy results.

		N	Adenoma	Hazard Ratio	*P*
**Overall**		490	147		
	<50yr	162	33	1 (Ref)	
	≥50yr	328	114	1.77 (1.21-2.60)	0.003**
**Overall**		490	147		
	G1	181	36	1 (Ref)	
	G2	27	3	0.71 (0.22-2.31)	0.570
	G3	105	36	1.75 (1.11-2.78)	0.017*
	G4	41	19	2.90 (1.66-5.06)	<0.001***
	G5	136	53	2.32 (1.52-3.54)	<0.001***
**<50yr**		162	33		
	G1	67	7	1 (Ref)	
	G2	16	1	0.74 (0.09-6.08)	0.773
	G3	26	9	3.89 (1.45-10.45)	0.007**
	G4	14	6	5.34 (1.78-15.98)	0.003**
	G5	39	10	3.33 (1.27-8.78)	0.015*
**≥50yr**		328	114		
	G1	114	29	1 (Ref)	
	G2	11	2	1.03 (0.25-4.34)	0.965
	G3	79	27	1.25 (0.74-2.11)	0.405
	G4	27	13	2.25 (1.17-4.33)	0.015*
	G5	97	43	1.89 (1.18-3.03)	0.009**
**G1**		181	36		
	<50yr	67	7	1 (Ref)	
	≥50yr	114	29	2.93 (1.28-6.72)	0.011*
**G2**		27	3		
	<50yr	16	1	1 (Ref)	
	≥50yr	11	2	1 (0-Inf)	1
**G3**		105	36		
	<50yr	26	9	1 (Ref)	
	≥50yr	79	27	0.86 (0.40-1.84)	0.697
**G4**		41	19		
	<50yr	14	6	1 (Ref)	
	≥50yr	27	13	1.11 (0.41-2.96)	0.838
**G5**		136	53		
	<50yr	39	10	1 (Ref)	
	≥50yr	97	43	1.71 (0.86-3.42)	0.128

*, **, and *** denotes P < 0.05, 0.01, and 0.001, respectively. G1 - G5, Group1 - Group5.

In the two age groups, the trend of the recurrence risk was similar, with some specific differences (**Table 3**). For patients <50 years old, the risks in the G3, G4, and G5 groups were significantly higher than that in the G1 group [HR, 3.89 (95% CI, 1.45–10.45), G3 vs. G1; HR, 5.34 (95% CI, 1.78–15.98), G4 vs. G1; and HR, 3.33 (95% CI, 1.27–8.79), G5 vs. G1], but the risk in G2 was not significantly different from that in the G1 group [HR, 0.74 (95% CI, 0.09–6.08), *P* = 0.773]. For patients ≥50 years old, the risks of recurrence in G4 and G5 groups were significantly higher than that in G1 group [HR, 2.25 (95% CI, 1.17–4.33), G4 vs. G1; HR, 1.89 (95% CI, 1.18–3.02), G5 vs. G1], but the differences in the risks in the G2, G3, and G1 groups were not significant [HR, 1.03 (95% CI, 0.25–4.34), *P* = 0.965, G2 vs. G1; HR, 1.25 (95% CI, 0.74–2.11), *P* = 0.405, G3 vs. G1].

**Figure 2:**
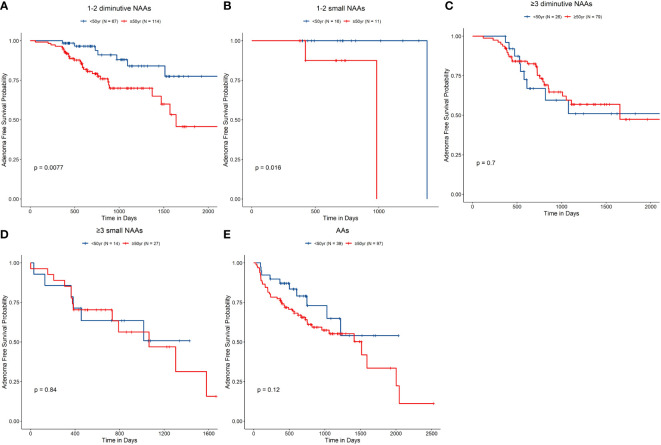
Non-advanced adenoma (NAA)–free survival rate according to the index colonoscopy results. **(A–E)**, G1–G5, accordingly. G1, Group 1, 1–2 diminutive NAAs; G2, Group 2, 3 or more diminutive NAAs; G3, Group 3, 1–2 small NAAs; G4, Group 4, 3 or more small NAAs; and G5, Group 5, advanced adenomas.

We next conducted subgroup analysis for age (≥50 years old vs. <50 years old) and the G1–G5 groups (**Table 3** and **Figure 2**). The analysis revealed that in the G1 group, the risk in the ≥50 years old group was remarkably higher than that in the <50 years old group [HR, 2.93 (95% CI, 1.28–6.72), *P* = 0.011] (**Figure 2A**). However, in other groups, the analysis did not reveal any significant differences by age (**Figures 2B–E**).

### 3.3 Risk of metachronous AN after resection of adenomas in different age groups

**Figure 3:**
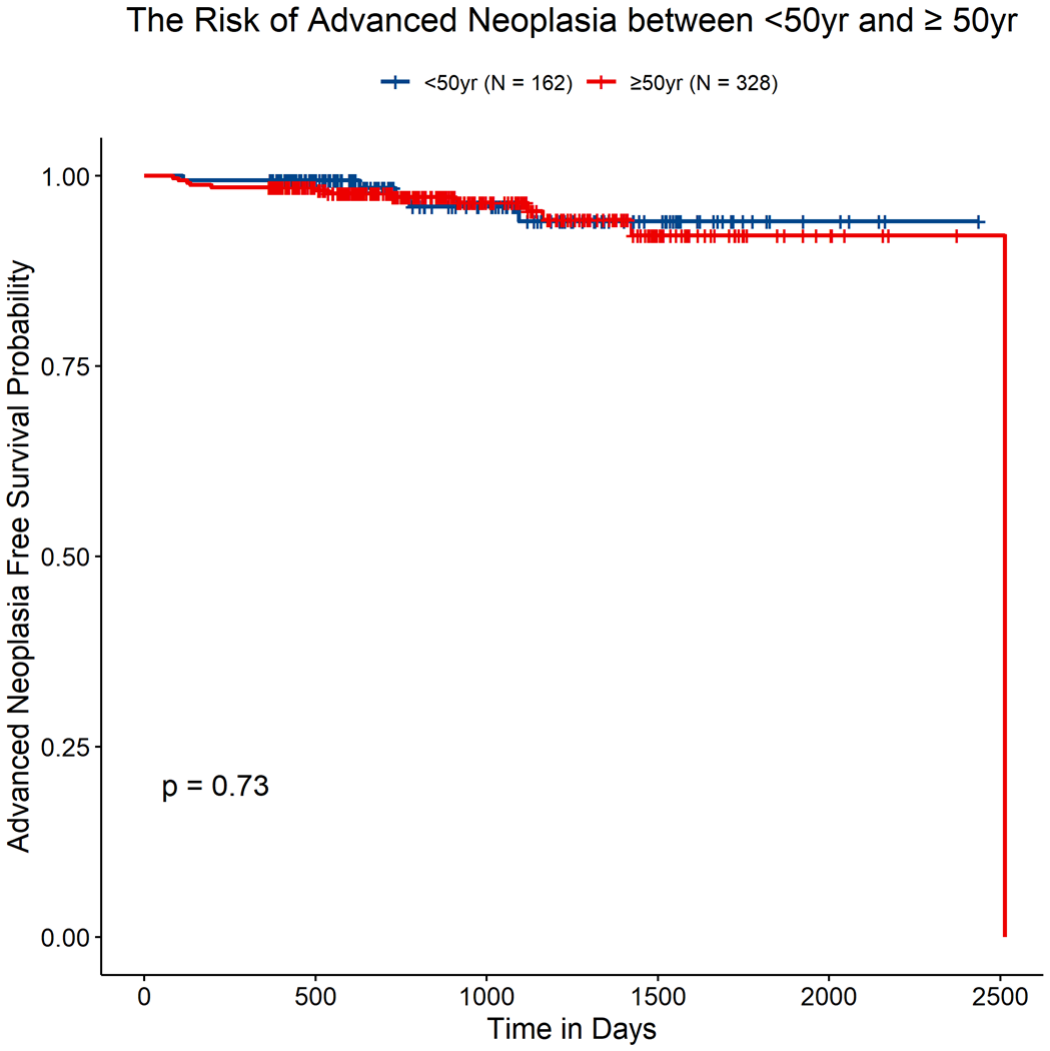
Advanced neoplasia-free survival rate in patients <50 years old and ≥50 years old.

Cumulative risks of metachronous AN in the different age groups are shown in **Table 4** and **Figure 3**. After adenoma resection, no differences in recurrence rates of AN [3.1% (n = 5) for <50 years old patients and 4.0% (n = 13) for ≥50 years old patients] were apparent in the two age groups [HR, 1.20 (95% CI, 0.42–3.41), *P* = 0.732). The general recurrence rate was 3.7% in all patients.

**Table 4:** Risk of metachronous advanced neoplasia between different groups.

		N	Advanced neoplasia	Hazard Ratio	*P*
**Overall**		490	18		
	<50yr	162	5	1 (Ref)	
	≥50yr	328	13	1.20 (0.42-3.41)	0.732
**Overall**		490	18		
	Without non-advanced adenoma recurrence	343	16	1 (Ref)	
	With non-advanced adenoma recurrence	147	2	0.25 (0.06-1.10)	0.066
**<50yr**		162	5		
	Without non-advanced adenoma recurrence	129	3	1 (Ref)	
	With non-advanced adenoma recurrence	33	2	1.92 (0.32-11.56)	0.477
**≥50yr**		328	13		
	Without non-advanced adenoma recurrence	214	13	1 (Ref)	
	With non-advanced adenoma recurrence	114	0	0 (0-Inf)	0.998

### 3.4 Risk of metachronous AN with NAA recurrence vs. without NAA recurrence

**Figure 4:**
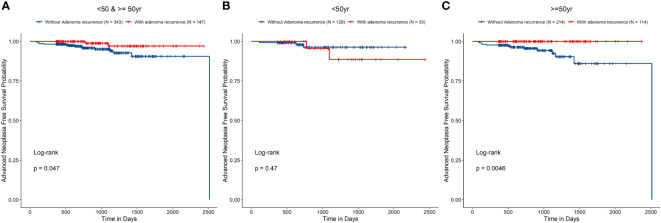
Risk of metachronous advanced neoplasia in groups (**A** All patients, **B** patients younger than 50 years old, and **C** patients 50 years old and over) with and without non-advanced adenoma recurrence.

Patients whom were found with polyp recurrence during surveillance would undergo polypectomy. To determine whether polyp recurrence, suggesting that these patients are more prone to metachronous AN, we compared the outcomes in patients with and without NAA recurrence in each age group. Cox regression analysis did not reveal significant differences in the risk of metachronous AN between patients with and without NAA recurrence [HR, 0.25 (95% CI 0.06–1.10), *P* = 0.066]. However, log-rank test of the survival curves of patients with and without NAA recurrence indicated significant differences between the groups (*P* = 0.047) (**Table 4** and **Figure 4A**). The differences were more pronounced for patients who were over 50 years old than for younger patients (**Figures 4B, C**). Log-rank test showed in patients ≥50 years old, the risk of metachronous AN was higher in patients without NAA recurrence than that of patients with NAA recurrence (*P* = 0.005), while in patients <50 years old this difference was insignificant (*P* = 0.47).

## 4 Discussions

In the current study, we compared the risk of NAA recurrence in two age groups (<50 years old and ≥50 years old) of patients with different numbers of different-sized adenomas. The presented data support the hypothesis that the NAA recurrence risk in patients with adenomas who are <50 years old is lower than that in patients ≥50 years old. Therefore, for patients with adenomas who are <50 years old, one may consider a different follow-up strategy than that for older patients.

The main results of the current study can be summarized as follows. (1) For patients <50 years old with 1–2 diminutive adenomas, the NAA recurrence risk after polypectomy is significantly lower than that for patients ≥50 years old with 1–2 diminutive adenomas. Hence, for these patients, one could recommend a surveillance interval that is longer than that recommended for patients ≥50 years old. (2) For patients <50 years old with small adenomas, the NAA recurrence risk is similar to that for patients ≥50 years old with small adenomas. Consequently, for these patients, the recommended surveillance frequency may be similar to that recommended for patients ≥50 years old.

Adenomatous polyps are typically considered to be a type of precancerous lesion before CRC (4) and most CRCs are thought to originate from adenomas. Removal of adenomas, once found, and subsequent surveillance are a standardized procedure in the US (5, 7). The current US Multi-Society Task Force guideline recommends stratifying patients ≥50 years old into different risk groups according to the baseline colonoscopy findings. According to the guideline, 1–2 NAAs that are <10 mm in size are considered low-risk adenoma, with a recommended 7–10-year surveillance interval for patients after polypectomy. Other adenomas are considered high-risk adenomas, with a recommended 3–5-year surveillance interval after polypectomy. However, the surveillance intervals recommended by the current guideline have some limitations and should be improved. Specifically, the guideline only provides recommendations for patients ≥50 years old, because of lack of research focusing on younger patients (<50 years old). Further, it does not discriminate between diminutive and small adenomas, even though some experts suggest that there are differences in the risk for these two types of adenomas to develop into malignant lesions (11, 14–17, 20). Similarly, the guidelines of European Society of Gastrointestinal Endoscopy (ESGE) and British Society of Gastroenterology (BSG) do not discriminate between diminutive and small adenomas and suggest a simpler follow-up protocol (23, 24). In ESGE and BSG guidelines, only adenomas ≥10mm, or with high grade dysplasia are regarded as high-risk adenomas, irrespective of villous components; others are all regarded as low-risk adenomas. ESGE guideline recommends that patients with 1-4 low-risk adenoma do not need any surveillance, while patients with ≥5 adenomas or with high-risk adenoma require surveillance colonoscopy in 3 years after index colonoscopy (23). BSG guideline is very similar with ESGE guideline, which recommends only patients with ≥2 premalignant polyps including ≥1 high-risk adenoma, or patients with ≥5 adenomas require surveillance colonoscopy in 3 years (24).

The risks of metachronous AN in patients who had undergone polypectomy of diminutive or small adenoma have been compared in several other studies (11, 14–17, 20). Although the endpoint chosen in the current study was different from those of the other studies, the conclusions presented herein are still generally in line with those of previous studies. In all studies, patients with 1–2 diminutive NAAs were at a lower risk of either metachronous AN or NAA recurrence than patients with 1–2 small NAAs. Sneh Arbib et al. (14) were the first to report that patients with 1–2 diminutive NAAs were at a different risk of metachronous AN than patients with 1–2 small NAAs. However, no differences in the risk in patients with ≥3 polyps were reported in that study (14). These observations were further validated in subsequent investigations worldwide (11, 15, 17, 20). Kim et al. (11, 20) validated these conclusions in Asian populations. The authors reported that while patients with high-risk adenoma (including ≥3 diminutive adenomas, ≥3 small adenomas, and AAs) shared a similar risk of metachronous AN, the risk was reduced in patients with 1–2 diminutive NAAs. Further, the risk was different for patients <50 years old and patients ≥50 years old. In patients <50 years old with high-risk adenoma, the risk was no longer similar to that of patients ≥50 years old with high-risk adenoma (20). The risk of metachronous AN in patients with ≥3 diminutive adenomas was lower than that in patients with ≥3 small adenomas, but was not different from that in patients with low-risk adenoma. Kim et al. (11) did not conduct any further subgroup analysis according to age in each group. Nonetheless, they concluded that the surveillance strategy should probably be different for patients <50 years old and ≥50 years old. Our study supported this notion, but with some specific cases. For example, we did not find any differences between the risk of NAA recurrence in patients with ≥3 diminutive adenomas between patients <50 years old and ≥50 years old [HR, 0.86 (95% CI, 0.40–1.84), *P* = 0.697].

Considering the above, the follow-up strategy for the surveillance interval should be updated to guide long-term prognostic assessment and follow-up. Jung et al. (12) proposed a new classification that entails three groups (low, intermediate, and high-risk groups) instead of two groups (low and high-risk groups). They suggested that 1–2 NAAs sized 6–9 mm and 3–10 NAAs sized 1–5 mm should be regarded as an intermediate-risk group and require a different surveillance interval than other groups. However, the authors did not recommend any specific surveillance intervals for the risk groups in their study. Similar to the observations of Jung et al. (12) for patients ≥50 years old, we here observed that 1–2 small NAAs are more dangerous than 1–2 diminutive NAAs in that patient group. In addition to showing that the same holds for patients <50 years old, we also found that even with just 1–2 diminutive NAAs, patients ≥50 years old are at a much greater risk of adenoma recurrence than patients <50 years old [HR 2.93 (95%CI 1.28–6.72)]. Therefore, an individualized surveillance strategy should be established that considers various factors, especially age.

We also here analyzed the risk of metachronous AN in patients with and without NAA recurrence. A notion exists in clinical practice that polyp removal always reduces a patient’s risk of colorectal cancer. However, there is little evidence on the degree of the associated risk reduction. The observations presented herein demonstrate that during surveillance, recurrent NAAs which were timely resected would not increase the chances of metachronous AN comparing to patients without NAA recurrence. This result provides indirect evidence for the protective effect of polypectomy.

Further, we did not observe a significant difference in the risk of metachronous AN in patients <50 years old and ≥50 years old. We suggest that the low incidence of AN and some confounding factors, such as subsequent polypectomy after an index colonoscopy, may have impacted this finding. Because of the low incidence of AN, the sample size in the current study was not sufficient for subgroup analysis with respect to the size and number of adenomas. Consequently, while we conclude that the general risk of metachronous AN in patients <50 years old and ≥50 years old is similar, the risk in each subgroup requires further consideration.

One potential confounding factor needed to be considered was that there might be a few polyps missed at the first index colonoscopy, then regarded as recurrent polyps at the subsequent colonoscopy. Missing adenoma could not be avoided, but since the colonoscopies were all done by experienced endoscopists in our center, and all the patients included in the study were with a fair bowel preparation quality, this influence could be minimized. Our study showed a similar polyp recurrence rate with previous researches (11, 14), which also proved this confounding factor had little effect on the finding of the study. In our study, we found that the overall non-advanced adenoma recurrence rate during a median follow-up of 2.18 years was 30%. And in similar researches, Sneh Arbib et al. reported that the overall non-advanced adenoma recurrence rate during a median follow-up of 2.67 years was 30.5% (14). Kim et al. reported that 3-year nonadvanced adenoma cumulative recurrence rates after polypectomy were 39.2% for high-risk population (high-risk referred to those having an advanced adenoma or >3 adenomas) in 50-54 age, and 38.8% for high-risk population in 20-49 age (11). And for advanced neoplasia, similar to our study, there are several studies have reported a similar advanced neoplasia recurrence rate. Kim et al. reported that the overall advanced adenoma recurrence rate during a median follow-up of 3.2 years was 5.6% (20). And in another study, Kim et al. reported that 3-year cumulative advanced adenoma recurrence rate was 0.9-4.0% varying from patients with different baseline adenoma numbers and sizes (13).

The current study has several limitations. First, the follow-up period was relatively shorter than that recommended by the current guideline but close to that used in similar studies. The average follow-up time is 2.52 years (median: 2.18 years), while the median follow-up time was 2.67 years for the study by Sneh Arbib et al. (14). Second, the sample size of some subgroups was inadequate to detect associations, such as the G2 group (n = 27) and G4 group (n = 41). However, the primary conclusions of the current study are not based on data for the G2 group and G4 group and, therefore, this limitation does not undermine the primary conclusions of the study. Since a small sample size may produce a false-negative error, the true correlations for the G2 group should be validated in a large-sample study. Third, the cohort in the current study was based on the medical records of Wuhan Union Hospital. The cohort was a hospital-based population, and we selected all patients and checkup populations who met the inclusion criteria in the study, which may have introduced selection bias. Further, the retrospective design of the study did not allow a constant follow-up duration. The time interval between the index colonoscopy and subsequent colonoscopy varied, which could also result in bias. Accordingly, we used the survival analysis method to minimize the effects of any such potential bias. Fourth, we found a slight difference on the mean ages of G1-G5 on the baseline. Thus, we conducted a subgroup analysis, divided the patients into the subgroups of <50 years old and ≥50 years old and found this heterogeneity was eliminated in each subgroup. So, this heterogeneity would not influence our major conclusions since our major conclusions were based on the subgroup analysis results. This also suggested the necessity to discriminate the patients <50 years old and ≥50 years old, which is the major topic of our research.

Several important aspects of the current study should be highlighted. To the best of our knowledge, the current study is the first to focus on the differences of adenoma recurrence risk in patients <50 years old and ≥50 years old. Some previous studies reported that the metachronous AN risk is different across age groups, but no further subgroup analysis for age was conducted (11, 20). Further, we chose NAA recurrence as the primary endpoint, which is a feasible endpoint in clinical practice. In conclusion, we showed that it is necessary to distinguish between patients ≥50 years old and <50 years old with 1–2 adenomas sized 1–5 mm because they are at a different risk of NAA recurrence, which may ultimately affect the risk of CRC development.

## Data availability statement

The data generated and analyzed during this study is included in the article. Further inquiries can be directed to the corresponding author on reasonable request.

## Ethics statement

The studies involving human participants were reviewed and approved by the Ethics Committee of Tongji Medical College, Huazhong University of Science and Technology (IORG number: IORG0003571). The patients/participants provided their written informed consent to participate in this study.

## Author contributions

SC and HS contributed equally to this work. RL designed and supervised the study and data analysis; SC and HS performed most of data collection, analysis, and wrote the manuscript. MF provided help in data collection and analysis. QZ provided pathological assessment and analysis. The authors read and approved the final manuscript.

## Funding

Supported by the National Natural Science Foundation of China (Nos. 81974068 and 81770539), Natural Science Foundation of Hubei Province (No. 2017CFA061) and the National key research and development program of China (No. 2017YFC0110003). The funding body had no part in the design of the study and collection, analysis, and interpretation of data and in writing the manuscript.

## Conflict of interest

The authors declare that the research was conducted in the absence of any commercial or financial relationships that could be construed as a potential conflict of interest.

## Publisher’s note

All claims expressed in this article are solely those of the authors and do not necessarily represent those of their affiliated organizations, or those of the publisher, the editors and the reviewers. Any product that may be evaluated in this article, or claim that may be made by its manufacturer, is not guaranteed or endorsed by the publisher.
